# From microbleeds to axonal damage: current evidence and future directions

**DOI:** 10.3389/fradi.2026.1760936

**Published:** 2026-03-13

**Authors:** Dávid Bognár, Zalán Petneházy, Péter Laár, Petra Bondor, Tamás Dóczi, Bálint Környei, Arnold Tóth

**Affiliations:** 1 Medical School, University of Pécs, Pécs, Hungary; 2 Department of Medical Imaging, University of Pécs, Pécs, Hungary; 3 National Laboratory of Translational Neuroscience, Pécs, Hungary; 4 Department of Neurosurgery, University of Pécs, Pécs, Hungary

**Keywords:** cerebral microbleeds, CMBS, diffusion tensor image (DTI), susceptibility weighted image (SWI), TAMVI, tractography, traumatic axonal injury (TAI), traumatic brain injury (TBI)

## Abstract

Traumatic brain injury (TBI) represents a major public health challenge worldwide. Traumatic axonal injury (TAI) is a key determinant of outcome yet remains difficult to assess directly *in vivo* in routine clinical practice and is therefore typically inferred indirectly using advanced neuroimaging techniques. Cerebral microbleeds (CMBs), detected primarily with susceptibility-based MRI sequences, have emerged as putative imaging markers associated with axonal injury and injury severity in TBI. A broad and heterogeneous body of literature has explored their relevance using gradient-echo (GRE), susceptibility-weighted imaging (SWI), quantitative susceptibility mapping (QSM), and diffusion tensor imaging (DTI), as well as neuropsychological correlates. In this mini review, we provide a focused narrative synthesis of studies examining the detection, characterization, and interpretative framework of CMBs in TBI, with particular emphasis on susceptibility-based MRI and diffusion imaging approaches. We highlight areas of convergence and inconsistency in the literature and discuss methodological and conceptual considerations relevant to the use of CMBs as context-dependent imaging markers in TBI.

## GRAPHICAL ABSTRACT

**Figure:**
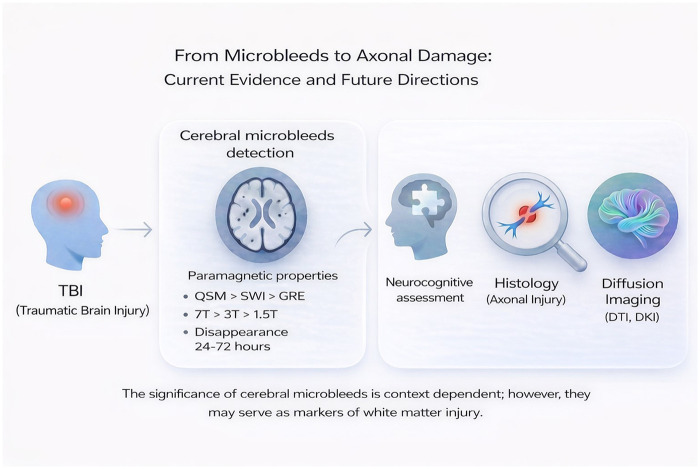
Overview of the proposed relationship between traumatic brain injury, cerebral microbleeds, and axonal damage, highlighting microbleed detectability and the multimodal assessment of white matter injury.

## Introduction

Traumatic brain injury (TBI) represents a major global public health challenge. Globally, 50–60 million people sustain a TBI each year, contributing to an estimated economic burden of nearly 400 billion USD and ranking TBI among the top three causes of injury-related death and disability projected through 2030 (1).

Accurate assessment of injury severity and prognosis is crucial for the optimal allocation of rehabilitation resources, ensuring both cost-effectiveness and equitable access to care. Numerous TBI rehabilitation strategies are under investigation, and their successful clinical implementation depends on precise and reliable diagnosis (2). Among the neuropathological consequences of TBI, traumatic axonal injury (TAI) plays a pivotal role in determining long-term outcomes. TAI results from the mechanical stretching and disruption of axons, predominantly affecting the white matter (WM) (3). Yet, despite its clinical significance, TAI often remains undetectable using conventional CT and standard MRI sequences. Advanced diffusion-based MRI techniques offer sensitive tools for detecting microstructural WM damage. However, their routine clinical use is limited by long acquisition times and computational complexity.

Importantly, certain pathological markers associated with TAI can be visualized. Among these, cerebral microbleeds (CMBs) have garnered growing interest. Because capillaries and adjacent axons occupy the same microanatomical milieu within the white matter, they are exposed to similar macroscopic mechanical loading during traumatic deformation. From a biomechanical perspective, large transient strains are therefore likely to affect both axons and the surrounding microvascular network, potentially resulting in axonal disruption and focal capillary injury in close spatial proximity. In line with this concept, the recently published nomenclature recommendation of the National Institute of Neurological Disorders and Stroke, introducing the term traumatic axonal and/or microvascular injury (TAMVI), underscores the close relationship between trauma-induced microvascular damage and axonal disruption (5). Accordingly, CMBs may co-occur with regions vulnerable to TAI and may serve as indirect, context-dependent radiological markers; however, this association is not uniform, and CMBs do not invariably reflect underlying axonal injury (4). But in some cases, CMBs may reflect isolated small-vessel pathology without relevant adjacent axonal disruption; therefore, not all CMBs imply clinically meaningful TAI (6).

Accordingly, CMBs can be conceptualized as mechanistic co-lesions associated with TAI, markers of global injury severity, or mixed vascular susceptibility lesions of heterogeneous origin, such as focal vessel wall fragility related to pre-existing microangiopathy or inflammation. Careful clarification of the conditions under which CMBs indicate concomitant TAI is therefore crucial. Establishing this distinction would support the use of CMB detection as a practical, context-dependent marker for TAI

### Detection of CMBs

Their detection relies on the magnetic susceptibility differences induced by paramagnetic blood degradation products relative to the surrounding diamagnetic brain tissue. These susceptibility effects form the basis of their appearance on T2*-weighted and susceptibility-weighted MRI (SWI) sequences (7), where CMBs typically appear as small, ovoid, or curvilinear hypointense foci with a maximum size up to 10 mm (8). In contrast, on quantitative susceptibility mapping (QSM), CMBs are characterized by positive magnetic susceptibility values (9)

The introduction of SWI, which incorporates both magnitude and phase information, has substantially improved the sensitivity of CMB detection (10). A previous study investigating subjects with CAA demonstrated that the use of clinical GRE sequences detected a total of 103 CMBs, whereas SWI identified 310 CMBs in the same three patients (11). Similarly, another study examining seven patients with TBI on MRI acquired on average five days after trauma reported that GRE detected 200 lesions using visual assessment and 162 lesions using computer-based measurement. In contrast, SWI identified substantially more lesions, with 935 detected by visual assessment and 1038 by computer-based measurement. Although SWI sensitivity for CMBs exceeds a T2- GRE technique, but the substantially longer acquisition time is a limiting factor (12). To address this, accelerated SWI techniques—such as 3D multishot and segmented echo-planar imaging (segEPI)—have been developed (13). Recent studies comparing conventional GRE-based SWI with segEPI-SWI in trauma populations found that the two methods perform comparably in detecting CMBs (14).

The detectability of CMBs is strongly influenced by the magnetic field strength. In a baseline cohort of 550 community-dwelling individuals, CMBs were detected in 45 participants at 3.0 T. Among the 25 individuals who underwent additional imaging at 1.5 T, a total of 53 CMBs were identified at 3.0 T compared with 41 at 1.5 T, corresponding to an approximately 30% higher detection rate at 3.0 T (15). Beyond conventional field strengths, ultra–high-field MRI further enhances CMB detection. In a cohort of 10 TBI patients examined on average 22 months after trauma, SWI identified 485 CMBs at 3 T, increasing to 584 CMBs (+20%) at 7 T with similar spatial resolution, and to 684 CMBs (+41%) at 7 T using tenfold higher spatial resolution (16, 17). However, the clinical utility of 7 T in TBI remains debated, Hütler et al. reported that its higher sensitivity did not translate into superior prognostic accuracy for indicators of the acute clinical state and chronic neurobehavioral parameters compared to 3 T imaging (18).

Radiological–histopathological correlation studies focusing on CMBs are available only in limited numbers, which constrains the interpretation and generalizability of their findings (19, 20). In a detailed postmortem correlation study, the mean interval between pre-mortem MRI and death was 18 months. Twenty-five cases underwent heterogeneous MRI protocols across scanners and field strengths (1.5 T and 3 T), while neuropathological analysis was performed corresponding to CMB locations. In total, 31 CMBs were identified on pre-mortem MRI, whereas histopathology detected an additional 25 lesions, resulting in 52 confirmed CMBs. Of the MRI-detected lesions, 27 were histopathologically confirmed, corresponding to a true-positive rate of 51.9%. Importantly, histopathology identified a substantial number of additional CMBs, yielding a false-negative rate of 48.1%. Given that histological analysis was restricted to slabs selected based on MRI findings, the true false-negative rate is likely underestimated. Four MRI-detected lesions represented false positives, primarily attributable to mimics (21). A brief summary is provided in Table 1, outlining etiological causes of CMBs as well as pathologies that mimic their appearance.

**Table 1:** Summary of the diverse etiological origins of cerebral microbleeds and the pathologies that may mimic their appearance on MRI.

Microbleeds etiologys			
Etiology	Typical clinical context	Characteristic distribution	Pathophysiological mechanism
Stroke (22)	Acute or subacute ischemic stroke	Peri-infarct and cortical–subcortical regions	Blood–brain barrier disruption and reperfusion-related microvascular injur
Traumatic brain injury (4)	Clinical history of TBI	Corpus callosum, brainstem, deep white matter, gray–white junction	Shear-induced capillary rupture adjacent to axonal injury
Hypertensive arteriopathy (23)	Chronic hypertension, older age	Deep gray nuclei (basal ganglia, thalamus), brainstem, cerebellum	Lipohyalinosis and rupture of small penetrating arteries
Cerebral amyloid angiopathy (CAA) (24)	Elderly, cognitive impairment	Lobar cortex and subcortical white matter	Amyloid-*β* deposition weakening vessel walls
aging (25)	Elderly patient	Mixed deep and periventricular distribution	Chronic microvascular degeneration
Radiation-induced vasculopathy (26)	Prior cranial radiotherapy	Within or adjacent to radiation field	Endothelial damage and delayed capillary fragility
Inflammatory vasculopathies (27)	Vasculitis, autoimmune disorders	Variable, often multifocal	Immune-mediated vessel wall injury
Antithrombotic Treatment/thrombolysis (28)	Anticoagulant therapy, bleeding disorders	Variable	Impaired hemostasis predisposing to microhemorrhage
Patient in critical condition (29, 30)	ECMO, ICU	Juxtacortical, corpus callosum	Hypoxia-induced hydrostatic and biochemical alterations can compromise blood–brain barrier integrity, erythrocyte extravasation
Fabry disease (31)	Lysosomal storage disorder	Variable	Vascular glycosphingolipid accumulation
Infective endocarditis (32)	Heart failure, heart murmur, inflamation	Pre dominantly cortical	Hypotheses: Pyogenic vasculitis or subacute microvascular inflammatory processes or endothelial dysfunction
Cardiac valve Replacement (33)	History of cardiac valve surgery	Variable	
Fat embolism (34)	Dyspnoe, neurologic symptoms, petechial hemorrhage and usualy fractures in history	Diffuse, small focal hypointensities	Starfield pattern, Walnut kernel pattern
Moyamoya (35)	Nonarterioscleotic, steno occlusive disease of the distal internal carotid arteries and their proximal branches,	Deep gray matter and periventricular regions	Chronic hemodynamic stress, fragile collateral vessels, and microvascular rupture
Microbleeds mimics			
Mimic	Imaging features	Key differentiating characteristics	
Calcifications (27)	Hypointense on SWI/GRE	Hyperdense on CT; diamagnetic on phase images	
Cavernous malformations (Type IV, Zabramski) (36)	Small punctate hypointensities	Often familial; associate with epileptic seisure or focal neurologic deficite	
Microaneurysms (37)	Focal susceptibility	May enhance or be visible on angiography	
Microthrombi (30)	Focal susceptibility	Often transient; may correlate with ischemic lesions	
Perivascular spaces (flow artifacts) (27)	Linear or dot-like signal loss	Follows vascular course; disappears on orthogonal planes	
Venous structures/venules (37)	Tubular hypointensity	Continuity across slices; orientation along veins	
Pneumocephalus (38)	Irregular susceptibility	Corresponds to intracranial air on CT; follows non-vascular spaces; atypical distribution; clinical history of trauma or surgery	
Iron deposits (basal ganglia) (39)	Symmetric hypointensity	Typical anatomical pattern; age-related	
Micrometastasis (40)	Hypointense on SWI/GRE	In the case of melanoma, post contrast T1 hyperintensiti	

Finally, the temporal dynamics of CMBs add further complexity to their interpretation. While many CMBs remain visible for years, animal models and human studies have demonstrated that some lesions can transiently disappear between 24 and 72 h post-injury (41, 42). Conversely, other reports have described a gradual increase in CMB size or conspicuity over time, suggesting evolving tissue responses at the injury site (43).

### Role of the CMB in the prognosis

Early investigations in boxers identified CMBs in 3 out of 42 (44) and 2 out of 21 (45) athletes, with none detected in control groups; however, these findings did not reach statistical significance. It is notable that while the first study used a 3 T MRI without SWI, the latter employed 1.5 T system, likely limiting sensitivity. Lawrence et al. using 3 T SWI detected CMBs in 6 of 13 TBI patients, with their presence associated with significantly lower initial Glasgow Coma Scale (GCS) scores. No CMBs were observed in patients with a GCS of 15, and reductions in CMB number correlated with GCS improvement over 15 days (46). Similarly, Tao et al. found a significant negative correlation between the number of SWI-detected CMBs and GCS scores in 25 patients (47). Other studies confirmed that patients with more severe TBI exhibit greater CMB burden, particularly in the corpus callosum, brainstem, and cerebral hemispheres. However, in binary logistic regression for the Glasgow Outcome Scale–Extended (GOSE), CMBs did not remain independent predictors—likely reflecting the lower sensitivity of 1.5 T imaging (48). A large multicenter study reported that the presence of CMBs was associated with lower GCS scores, higher TBI severity classification, and poorer GOSE outcomes (49). Beyond global outcomes, CMBs have also been associated with specific cognitive and psychiatric domains, although findings remain heterogeneous. In a cohort of 26 patients with mTBI and detectable CMBs on 3 T SWI (mean imaging time: 24.8 days post-injury), lower digit span performance was observed compared with patients without CMBs (50). In contrast, other studies reported no significant differences in working memory assessed 3–24 months post-injury using 3 T SWI in 77 service members with mTBI (51). Notably, in a prospective study, CMB burden in the frontal, parietal, and temporal lobes was significantly higher among 28 patients with mTBI, normal CT and conventional MRI, who developed depression at 1-year follow-up, compared with 137 patients without depressive symptoms; MRI was performed between 2 h and 3 days after injury (52). The heterogeneity observed across the literature likely reflects the inherently complex and multifactorial nature of the condition. However, the use of diverse neuroradiological modalities and modeling approaches may facilitate a deeper understanding of the role of CMBs.

### CMB linked to white matter vulnerability

A biomechanical finite element (FE) model has been applied to estimate the occurrence and distribution of CMBs. In this study, high-resolution 3 T and 7 T MRI QSM images were used to map the venous system and refine the FE model. The results showed that regions corresponding to CMBs exhibited high axial strain and elevated strain rates. Region-of-interest analysis of WM tracts showed significantly higher 95th percentile peak axial strain and strain rate in tracts containing CMBs compared to those without (axial strain: 0.197 vs. 0.163; strain rate: 64.9 vs. 57.0 s^−1^), indicating preferential localization of CMBs to the upper tail of the mechanical load distribution in the FE model (53). Beyond the development of CMBs, such strain potentially also causes substantial disruption to the WM architecture, leading to TAI. While SWI provides spatially detailed information on CMBs, diffusion tensor imaging (DTI) offers complementary insight into the microstructural consequences of TAI. Combining these two modalities Casson et al. examined 45 retired NFL players, applying global WM masks segmented with SPM8. Their findings indicated that SWI-detected CMBs and FA were associated with the number of “dings” (i.e., subconcussive head impacts) sustained during football play (54). Andersen et al. studied 14 patients with severe TBI in the subacute phase using 3 T SWI and subdivided the brain into five anatomical regions. A significant association between DTI-derived metrics and the presence of CMBs was observed only in the mid-sagittal region. In addition, *post hoc* predictive modeling showed that combining CMB burden with DTI parameters from deep brain regions was associated with improved prediction of Coma Recovery Scale–Revised (CRS-R) scores. While each modality alone demonstrated predictive value, the combined model showed the strongest correspondence with individual CRS-R scores (55).

Similarly, Aline et al. found that mild TBI (mTBI) patients with persistent CMBs at one year displayed significantly altered DTI metrics on tract-based spatial statistics (TBSS). In their study of 30 mTBI patients, those with CMBs and without showed significant group differences in eight neuropsychological test results at one week, with five of these assessing psychomotor speed and information processing speed. At 3 months, one test showed a significant difference, and at 1 year, five test results differed significantly, with the CMBs group performing worse in all but one measure. Additionally, the CMBs group reported higher symptom severity on the Post-Concussion Symptom Scale at 1 year, particularly for total symptoms, fatigue, difficulty concentrating, and difficulty remembering. The number of CMBs at 1 week correlated positively with ten symptom measures at 3 months and six at 1 year, indicating that a higher initial CMB burden was associated with greater subsequent symptom severity (56). In line with these findings, Haberg et al. reported that, among 73 moderate-to-severe TBI patients (according to the Head Injury Severity Scale) more than one-year post-injury, those who were CMB-positive showed global FA reductions and mean diffusivity (MD) increases in TBSS compared with CMB-negative patients (57). Dahl et al. also applied TBSS analysis, complemented by whole WM and atlas-based assessments. Identifying significant correlations between total CMB load and DTI metrics; however, these associations disappeared after adjusting for GCS or post-traumatic amnesia (PTA), suggesting that CMBs may partly reflect overall injury severity (58). Andreasen et al. further highlighted that the *spatial distribution* of CMBs carries valuable information: CMBs in deep midline regions (thalamus, basal ganglia) were associated with longer PTA durations and combining CMB count with FA improved the prediction of PTA length compared to FA alone in the subacute stage after severe TBI (59). In support of this, Mazwi et al. reported that CMBs in the hippocampus and corpus callosum were linked to prolonged PTA in univariate analysis. While admission GCS was the only individual predictor in multivariate analysis, a combination of hippocampal and callosal CMBs, age, and GCS together in regression tree analysis explained 26% of PTA variance and identified a subgroup with extended PTA (60). Similarly, Tóth et al. reported that the presence of any CMBs in the basal ganglia region was indicative of severe diffuse WM damage, even after adjusting for clinical variables and CT findings (61). Bognár et al. reported that in moderate–severe TBI (according to the Mayo classification), patients with CMBs showed greater DTI alterations than CMB-negative patients using TBSS. Whole-WM and tractography-defined WM DTI metrics correlated with CMB number, especially in the corpus callosum (62). The importance of the corpus callosum is highlighted by the independent association between callosal MD z-scores and CMB density in connected structures in moderate-to-severe TBI (63).

### Impact of CMBs on tractography

Diffusion measures acquired at 1 week and 6 months post-injury using unscented Kalman filter two-tensor tractography with whole-brain seeding showed that CMBs independently predicted FA decreases at 6 months, particularly in the corpus callosum, middle longitudinal fasciculus, and superficial temporal regions (64). Similarly in mTBI patients, Rostovsky et al. conducted a 6-month longitudinal follow-up study using deterministic tractography. They observed that even small CMBs, including those located deep within the brain, were associated with significant reductions in FA in surrounding WM tracts. To further explore these structural changes, they applied streamline matching to assess alterations in the trajectories of WM pathways over time. Using a prototype streamline calculation method to compute a curve index, they quantified changes in tract curvature and demonstrated that the trajectories of several WM pathways had undergone measurable alterations associated with the presence of CMBs (65). These findings may help explain why Petneházy et al. did not observe significant diffusion parameter differences when analyzing entire tracts in patients with moderate-to-severe TBI (according to Mayo). It is likely that when all voxels along a tract are averaged, focal alterations become diluted, masking localized abnormalities (66). Complementing these group-level findings, Maher et al. provided case-based evidence illustrating how individual CMBs can locally disrupt WM architecture in patients with mTBI. In the first patient CMB was located near the splenium of the corpus callosum. The perilesional fiber tracts showed asymmetric and divergent trajectories ipsilateral to the CMB, visible in both acute and chronic scans. In the second patient CMB was located near a fiber bundle connecting the right temporal and parietal lobes, and a post-injury shift of the tract toward the lateral ventricle was observed over time. Together, these observations demonstrate that even small CMBs may induce localized changes in surrounding WM and disrupt neural network circuitry, potentially contributing to long-term alterations in brain connectivity (67). Extending this to a larger cohort, Irimia et al. found that ∼97% of CMBs were surrounded by areas of significantly reduced FA (mean decrease: 34% ± 11%), particularly within associative tracts such as the corpus callosum, cingulum, and longitudinal fasciculi. These structural changes correlated with cognitive decline, with stronger effects in older and male patients (68).

### Local significance of CMBs

Corroborating these findings at a more localized level, Moen et al. investigated FA values in the immediate vicinity of CMBs using manually delineated masks. Their study revealed significantly lower FA values in patients compared to controls, with the reduction being even more pronounced for CMBs located within the corpus callosum (69). It is important to note that, in contrast to the findings of Griffin et al., who reported no histopathological correlation between CMBs and TAI (49), but the local significance of CMBs was further supported by Keene et al. They identified multiple CMBs in bull raiders with a history of TBI. MRI-guided postmortem histopathological analysis revealed signs of axonal necrosis in the corresponding regions, directly linking CMBs to localized TAI (70). One potential explanation for this discrepancy is that Griffin et al. focused on perilesional white matter surrounding chronic CMBs, where microstructural alterations may have been reversible and therefore no longer detectable at the chronic time point. In addition, not all CMBs necessarily colocalize with TAI, which may further contribute to the observed differences. This interpretation is further supported by the work of Mittenzwei et al., who analyzed postmortem brain from three donors with a history of severe TBI and death occurring 1–2 weeks after trauma. In their study, TAI—defined by the presence of APP-positive axonal swellings—was associated with 28 of 44 microbleeds (64%). This proportion was significantly higher than that observed in randomly sampled white matter regions (5 of 44 regions), suggesting a non-random spatial relationship between CMBs and TAI during the subacute phase following severe TBI (71).

### Future directions

A promising future direction is to more fully integrate QSM into CMBs research, given its superior ability compared to SWI to quantify (9). As Eskreis-Winkler et al. emphasize, QSM enables direct voxel-wise measurement of magnetic susceptibility making it more specific and quantitative than conventional SWI methods (72).

The standardization of CMB detection holds considerable potential for future research, particularly through the development of automated detection and characterization algorithms. While some semi-automated methods are already available (73), new automated methods not only promise to increase reproducibility and throughput across studies but also to extract clinically relevant metrics. Recent work has extended beyond binary lesion maps to automatically characterize CMBs by their size and anatomical distribution (74).

To accelerate tractography processing, convolutional neural network (based tractography approaches are already available, offering substantially faster reconstruction of white matter pathways compared to conventional diffusion-based algorithms (75). In addition, the application of diffusion kurtosis imaging and its integration with CMB assessment may offer valuable opportunities for capturing non-Gaussian diffusion effects and characterizing microstructural complexity associated with TAI.

Importantly, TBI is a multidimensional pathology, and many reported associations are context-dependent. Recent consensus frameworks have emphasized the need for multidomain assessment. Within such a framework, the role of CMBs may be better delineated, including the conditions under which they meaningfully reflect underlying white matter injury in conjunction with clinical severity and other biomarkers (5).

## Conclusion

In summary, available observational evidence suggests that CMBs are associated with TAI and injury severity, potentially reflecting both focal white matter disruption and more global network impairment. Their presence, burden, and spatial distribution have been linked to clinical, functional, and cognitive outcomes after TBI, although findings remain heterogeneous and context dependent. Importantly, CMBs' detectability and apparent burden depend on imaging sequence, timing after injury, and potential pre-existing microangiopathy. Further research is therefore needed to define the context-specific role of CMBs in clinical settings. In this regard, the adoption of the recently proposed TAMVI nomenclature is recommended in clinical practice to more accurately describe the underlying pathology.
